# Factors affecting neonatal mortality in the general population: evidence from the 2016 Ethiopian Demographic and Health Survey (EDHS)—multilevel analysis

**DOI:** 10.1186/s13104-019-4668-3

**Published:** 2019-09-23

**Authors:** Haileab Fekadu Wolde, Kedir Abdela Gonete, Temesgen Yihunie Akalu, Adhanom Gebreegziabher Baraki, Ayenew Molla Lakew

**Affiliations:** 1 Department of Epidemiology and Biostatistics, Institute of Public Health, College of Medicine and Health Sciences, University of Gondar, Gondar, Ethiopia; 2 Department of Human Nutrition, Institute of Public Health, College of Medicine and Health Sciences, University of Gondar, Gondar, Ethiopia

**Keywords:** Neonate, Mortality, EDHS, Factors

## Abstract

**Objective:**

This study was aimed to identify factors affecting neonatal mortality in Ethiopia.

**Results:**

According to the multilevel multivariable logistic regression analysis, the odds of neonatal mortality was significantly associated with husbands with no education (AOR = 2.30, 95% CI 1.10, 4.83), female birth (AOR = 0.57, 95% CI 0.39, 0.83), twin birth (AOR = 13.62, 95% CI 7.14, 25.99), pre-term birth (AOR = 15.07, 95% CI 7.80, 29.12) and mothers with no antenatal care (ANC) visit during pregnancy (AOR = 1.90 95% CI 1.11, 3.25).

## Introduction

Newborn is defined as an infant in the first 28 days of life after birth and newborn health has an important role in child’s survival and health. Globally, newborn deaths account for 45% of under-five deaths [1]. Three-quarters of newborn deaths result from three preventable and treatable conditions including prematurity, events during childbirth and neonatal infections [2, 3].

In 2017 alone, an estimated 6.3 million children and young adolescents died, mostly from preventable causes. Of all, about 2.5 million deaths occurred before celebrating their 28th days. Among children and young adolescents, the risk of dying was highest in the first month of life with average rate of 18 deaths per 1000 live births [4–6]. Despite there is an increase in neonatal mortality across the globe, its burden is highest in West and Central Africa, where the risk of a baby dying within the first 28 days of life is almost 10 times higher than high-income countries [7].

According to the United Nations (UN) mortality estimate in 2013, the neonatal mortality rate in Ethiopia was 28 per 1000 live births. Even though there is an achievement observed in the reduction of neonatal mortality by 48%, still neonatal mortality is high [8]. Additionally, according to the Ethiopian Demographic Health Survey (EDHS) 2011, it ranges from as low as 53/1000 live births in Addis Ababa to as high as 169/1000 live births in Benishangul-Gumuz region [8, 9].

According to different studies suggested there are many factors contributing to neonatal mortality. Among these: educational level [10], sex of the neonate, duration of pregnancy [11], home delivery without skilled provider [12], pregnancy complication [13], birth weight [14, 15], delay in seeking care during illness [16, 17], lack of preparedness of families and care providers, harmful cultural practices [18], economic status [19], social exclusion, maternal illiteracy [20], negative parental attitudes arising from the social environment, gender bias, inability to pay for care [20], and lack of basic prenatal, natal, and postnatal services [7, 21] were the main determinants of poor newborn survival rates in developing countries [22–26].

In 2014, at the Sixty-seventh World Health Assembly, 194 member states of the WHO develop action plan that was targeted to end preventable deaths and stillbirths [19]. Similarly, Ethiopia has endorsed strategies to halt the burden of neonatal mortality through ANC, postnatal care (PNC), immunization during and after pregnancy, and skill birth attendance. Despite there is a reduction in neonatal mortality, still there is a need to have focused attention on newborn interventions [10]. Therefore, the aim of this study was to identify factors affecting neonatal mortality in Ethiopia.

## Main text

### Methods

Community-based cross-sectional study was conducted in Ethiopia from January 18 to June 27, 2016. There are nine regional states and two city administrations subdivided into 68 zones, 817 districts and 16,253 kebeles (lowest local administrative units of the country).

The target group for this study was all neonates in Ethiopia, and those neonates in the selected enumeration areas (EA) were the study population. Stratified two stage cluster sampling was performed. Samples of EAs were selected independently in each stratum in two stages. Firstly, a total of 645 EAs (202 in urban and 443 in rural areas) were selected with probability sampling proportional to EA size and secondly, a fixed number of 28 households per cluster were selected randomly. A total of 18,008 households were randomly selected, and 15,683 eligible women were interviewed. Data on 10,641 live births were extracted from 2016 EDHS.

Secondary data source from 2016 EDHS was used. Approval letter was obtained from the measure DHS and the data set was downloaded from the DHS website (http://www.measuredhs.com).

The outcome variable for this study was neonatal death which was defined as the death of a live birth within the first month of life. Individual level factors included in this study were; current age of the mother, marital status, educational status both for the mother and the father, mothers occupational status, size of child at birth, child sex, birth order, duration of pregnancy, preceding birth interval and weather the child is twin or not, place of delivery, number of ANC visits, number of tetanus toxoid (TT) injections during pregnancy, mode of delivery and wealth index. Household wealth index was originally classified into five categories by DHS which was done using principal component analysis. However, in this study we further classified it into poor, medium, and rich. Community level factors included in this study were residence and region. Training was given for data collectors and supervisors and the questioner was pretested.

After data was extracted editing, coding and cleaning were performed. Both descriptive and analytic statistic was computed. Since the data had hierarchical and clustering nature, mixed effect logit model (multilevel model) was fitted to identify factors associated with neonatal death. Due to the fact that the rate of neonatal death varies from cluster to cluster, a cluster level random intercept was introduced in the mixed logit model. The within cluster correlation was measured using intra cluster correlation (ICC) which is expected to be ≥ 10% to use the model. To test the significance of the variance of random intercept, the likelihood ratio test was applied. Adjusted odds ratio with 95% confidence level was reported to show the strength of the association and its significance. Variables having p-value < 0.05 was considered as having significant association with the outcome. The model goodness of fit was checked using deviance information criteria (DIC). The data was analyzed using Stata Version 14.

### Results

#### Socio demographic and economic characteristics of mothers

A total of 10,641 live births were included in this study. Of these, 8667 (81.45%) were from rural residence and 1581 (14.86%) were from Oromia region. Majority 3161 (29.7%) of the mothers were in the age group of 25–19 years. More than half 5775 (54.3%) of the households were in a poor wealth quintile. Around 6838 (64%) of the mothers and almost half the husbands had no formal education (Table 1).

**Table 1:** Socio demographic and economic characteristics of participants, EDHS 2016

Variable	Un-weighted frequency (%)	Weighted frequency (%)
Age		
15–19	404 (3.80)	378 (3.43)
20–24	2171 (20.40)	2068 (18.76)
25–29	3161 (29.71)	3353 (30.42)
30–34	2360 (22.18)	2489 (22.58)
35–39	1697 (15.95)	1772 (16.08)
40–44	647 (6.08)	723 (6.56)
45–49	201 (1.89)	240 (2.17)
Residence		
Urban	1974 (18.55)	1216 (11.03)
Rural	8667 (81.45)	9807 (88.97)
Mothers educational status		
No education	6838 (64.26)	7284 (66.08)
Primary	2678 (25.17)	2951 (26.77)
Secondary and above	1125 (10.57)	788 (7.15)
Husbands educational status (n = 10,008)		
No education	5003 (49.99)	5077 (48.53)
Primary	3220 (32.17)	4116 (39.34)
Secondary and above	1785 (17.84)	1270 (12.14)
Mothers occupation		
Housewife	6307 (59.27)	6127 (55.58)
Employed	4334 (40.73)	4896 (44.42)
Marital status		
Currently married	9903 (93.06)	10,339 (93.79)
Currently unmarried	738 (6.94)	684 (6.21)
Wealth index		
Poor	5775 (54.27)	5156 (46.78)
Medium	1466 (13.78)	2280 (20.68)
Rich	3400 (31.95)	3587 (32.54)
Religion		
Orthodox	3082 (28.96)	3772 (34.22)
Catholic	72 (0.68)	103 (0.94)
Muslim	5442 (51.14)	4561 (41.38)
Protestant	1862 (17.50)	2329 (21.13)
Traditional	103 (0.97)	150 (1.36)
Other	80 (0.75)	107 (0.97)
Region		
Tigray	1033 (9.71)	716 (6.49)
Afar	1062 (9.98)	11.4 (1.04)
Amhara	977 (9.18)	2072 (18.80)
Oromia	1581 (14.86)	4851 (44.01)
Somali	1505 (14.14)	508 (4.61)
Benshangul	879 (8.26)	122 (1.10)
SNNPR	1277 (12.0)	2296 (20.83)
Gambela	714 (6.71)	27 (0.24)
Harari	605 (5.69)	26 (0.23)
Addis Ababa	461 (4.33)	244 (2.21)
Dire Dawa	574 (5.14)	47 (0.43)

#### Child characteristics and maternal health service use

Of 10,641 live births, more than half 5483 (51.5%) were male. Of the total live births, 3338 (31.4%) were in birth order 2nd–3rd and 10,455 (98.3%) were term births. The preceding birth interval for the majority 6356 (59.7%) of live births were 2 years and above. From a total of 7193 mothers of a new born, majority 4712 (65.5%) had at least one ANC visit during their pregnancy (Table 2).

**Table 2:** Maternal health service use and child characteristics of participants, EDHS 2016

Variable	Un-weighted frequency (%)	Weighted frequency (%)
Size of the child at birth		
Large	3214 (30.20)	3485 (31.62)
Average	4419 (41.53)	4580 (41.55)
Small	2890 (27.16)	2866 (26.00)
Don’t know	118 (1.11)	92 (0.83)
Birth order		
1st	2167 (20.36)	2058 (18.67)
2nd–3rd	3338 (31.37)	3359 (30.47)
4th–6th	3385 (31.81)	3603 (32.69)
≥ 7	1751 (16.46)	2003 (18.17)
Child sex		
Male	5483 (51.53)	5725 (51.94)
Female	5158 (48.47)	5298 (48.06)
Child is twin		
Yes	278 (2.16)	292 (2.65)
No	10,363 (97.39)	10,730 (97.35)
Duration of pregnancy		
Term	10,455 (98.25)	10,842 (98.36)
Pre term pregnancy	186 (1.75)	181 (1.64)
Preceding birth interval		
Not applicable	2167 (20.36)	2058 (18.67)
< 2 years	2118 (19.90)	1942 (17.62)
≥ 2 years	6356 (59.73)	7023 (63.71)
Delivery by CS		
Yes	305 (2.87)	213 (1.93)
No	10,336 (97.13)	10,810 (98.07)
Number of TT injections during pregnancy (n = 7193)		
0–1	4203 (58.43)	4473 (58.94)
≥2	2990 (41.57)	3117 (41.06)
Number of ANC visits during pregnancy (n = 7193)		
No visit	2481 (34.49)	2818 (37.13)
1–3 visit	2092 (29.08)	2343 (30.86)
≥ 4 visit	2620 (36.42)	2430 (32.01)

*CS* cesarean section, *TT* tetanus toxoid, *ANC* antenatal care

#### Factors associated with neonatal mortality

After excluding 32.4% missing data, the multivariable multilevel model was fitted and found ICC of 11% (95% CI 6.2, 18.6) and deviance of 1167.68. From the final model, being born from a mother whose husband had no education increase the odds of neonatal death by 2.30 (AOR = 2.30, 95% CI 1.10, 4.83) times compared to husbands with secondary education and above education. Being born from a mother whose husband had primary education increases the odds of neonatal death by 2.85 (AOR = 2.85, 95% CI 1.38, 5.90) times as compared to husbands with secondary education and above. The odds of neonatal death was decreased by 43% (AOR = 0.57, 95% CI 0.39, 0.83) among female births compared to their counterparts. The odds of neonatal death was 13.62 (AOR = 13.62, 95% CI 7.14, 25.99) times higher among twin births as compared to singleton. The odds of neonatal death was 15.07 (AOR = 15.07, 95% CI 7.80, 29.12) times higher among pre term births compared to term. The odds of neonatal death was 1.9 (AOR = 1.90 95% CI 1.11, 3.25) times higher for births to mother who didn’t have ANC during pregnancy compared to mothers who had ≥ 4 ANC visits (Table 3).

**Table 3:** Multilevel multivariable logistic regression output for determinants of neonatal mortality among live births in Ethiopia, 2016

Variable	Null model	Model II AOR (95% CI)	Model III AOR (95% CI)	Model IV AOR (95% CI)
Age				
15–19		1		1
20–24		1.13 (0.41, 3.14)		1.16 (0.42, 3.21)
25–29		1.17 (0.40, 3.36)		1.21 (0.42, 3.52
30–34		0.71 (0.23, 2.23)		0.75 (0.24, 2.34
35–39		1.33 (0.43, 4.05)		1.37 (0.45, 4.22)
40–44		1.94 (0.60, 6.30)		2.00 (0.62, 6.54)
45–49		3.45 (0.96, 12.43)		3.54 (0.98, 12.78)
Residence				
Urban			1	1
Rural			1.84 (1.26, 2.67)	1.31 (0.68, 2.52)
Husband’s educational status				
No education		2.54 (1.25, 5.14)		2.31 (1.10, 4.83)*
Primary		3.10 (1.54, 6.25)		2.85 (1.38, 5.90)**
Secondary and above		1		1
Child sex				
Male		1		1
Female		0.57 (0.39, 0.83)		0.57 (0.39, 0.83)**
Child is twin				
No		1		1
Yes		13.63 (7.15, 26.00)		13.62 (7.14, 25.99)***
Duration of pregnancy				
Term		1		1
Pre term		14.61 (87.60, 28.07)		15.07 (7.80, 29.12)***
Preceding birth interval				
Not applicable (single birth)		1.42 (0.76, 2.67)		1.46 (0.77, 2.75)
< 2 years		1.51 (0.96, 2.37)		1.50 (0.96, 2.36)
≥ 2 years		1		1
Number of TT injections during pregnancy (n = 7193)				
No or one injection		1.01 (0.64, 1.59)		1.00 (0.64, 1.59)
≥ 2 injections		1		1
Number of ANC visit during pregnancy (n = 7193)				
No visit		2.00 (1.18, 3.38)		1.90 (1.11, 3.25)*
1–3 visit		0.95 (0.56, 1.62)		0.92 (0.53, 1.57)
≥ 4 visit		1		1
Intercept	0.025 (0.022, 0.030)	0.004 (0.0019, 0.0090)	0.015 (0.011, 0.022)	0.004 (0.002, 0.008)
Log-likelihood	− 1428.86	− 584.26	− 1423.19	− 583.84
Deviance	2857.72	1168.52	2846.38	1167.68

*** *p*-value < 0.001, ** *p*-value < 0.01, * *p*-value < 0.05

### Discussion

This study was conducted among neonates in Ethiopia. Accordingly husband education, child sex, child twin, duration of pregnancy, and number of ANC visit were significantly associated with neonatal death.

The odds of neonatal death was higher among births to mother whose husband had no education and primary education compared to husbands with secondary education and above. This finding was in line with the study conducted in northwest Ethiopia [10]. This could be husbands having secondary and above level of education resulted in better knowledge of solutions and critical decisions during crisis. In addition, better education creates opportunity for better economic status [10].

The odds of neonatal death was lower among female births compared with male births. This finding is in agreement with a study from Ghana [11], Nigeria [27], and Indonesia [28]. Accordingly, male neonates were at higher risk of death than female neonates. This could be attributed by the different protein and gene expression in both male and female fetuses due to variation in placenta especially during adverse condition. Besides, the same extracellular micro RNA may show up regulation in females and down regulation in male fetuses within the intrauterine milieu. These makes females to have a natural survival advantage than males [29].

The odds of neonatal death was higher among twin births compared with singleton births. This study was supported by a study conducted in Korea [30]. The possible explanation could be twin pregnancy is usually associated with prematurity, which is the most common cause of neonatal death and twin-to-twin transfusion syndrome which further leads to death [31]. In addition, twin pregnancy is usually end up with low birth weight which increase child vulnerability to infection and decreases their immunity [27]. As a result, child survival is decreased. This study was also consistent with a study done from 60 nationally-representative Demographic and Health Surveys data [32].

In this study, preterm birth was associated with higher odds of neonatal death compared with term pregnancy. This finding was in agreement with a study done in Ghana [11], Ethiopia [17, 33], and Indonesia [28]. This could be the fact that preterm neonates were unable to fit the extra uterine life because of poor lung maturation, resulted in unable to breathe and hypoxia, ends with death [34]. Therefore, during preterm birth to enhance fetal lung maturation betamethasone is recommended [35]. Besides preterm neonates were at higher risk of vitamin D deficiency, which resulted in respiratory distress [36] and further end up with death.

Being born from a mother with no ANC visit increase the odds of neonatal death compared to mothers with ≥ 4 ANC visits. This result was supported by the study conducted in Nigeria [27], Sub-Saharan Africa [21], and Iran [37]. The possible reason could be women having ANC visit have a chance of prompt detection of complication and early initiation of breastfeeding, which boost the immunity of a child [36, 38]. In addition, women who had complete ANC follow up had increased probability of giving birth by skilled birth attendant, which decreases the odds of neonatal death [37]. Moreover, ANC follow up usually leads to have quality essential new born cares, which increase neonatal survival [39].

This study has both public and clinical importance. For the public it gives direct information regarding the possible cause of neonatal death and alerts them to take the possible appropriate interventions. In addition, identifying determinates of mortality gives information for clinicians to take prompt intervention and response to halt the high burden of neonatal death.

### Conclusion

In this study husband with no or primary education, being male neonate, being twin birth, preterm pregnancy, and having no ANC visit were statistically significant predictors of neonatal death. Therefore, women should be encouraged to have ANC visit. Close follow up and monitoring should also be given for twin and preterm births.

## Limitation

This study shares the limitation of cross-sectional study to create temporal relationship between the exposure and the outcome variable. This study might also be affected because of residual confounding factors that are not assessed in this study and misclassification of variables like size of the child at birth.

## Data Availability

Data is available on https://dhsprogram.com/data/available-datasets.cfm.

## Abbreviations

ANC: antenatal care
AOR: adjusted odds ratio
ARR: annual reduction rate
CI: confidence interval
CS: cesarean section
DIC: deviance information criteria
EA: enumeration area
EDHS: Ethiopian Demographic Health Survey
ICC: intra cluster correlation
PNC: postnatal care
SDG: sustainable development goal
SSA: Sub-Saharan Africa
TT: tetanus toxoid
UN: United Nations
WHO: World Health Organization
